# Effect of Mucosal Brushing on the Serum Levels of C-Reactive Protein for Patients Hospitalized with Acute Symptoms

**DOI:** 10.3390/medicina56100549

**Published:** 2020-10-19

**Authors:** Naoko Nakahodo, Yoshiaki Nomura, Takumi Oshiro, Ryoko Otsuka, Erika Kakuta, Ayako Okada, Yuko Inai, Noriko Takei, Nobuhiro Hanada

**Affiliations:** 1 Okinawa Kyodo Hospital, 4-10-55 Kohagura, Naha, Okinawa 900-8558, Japan; dh_nakahodo_naonao219@yahoo.co.jp; 2 Department of Translational Research, School of Dental Medicine, Tsurumi University, Yokohama 2308501, Japan; otsuka-ryoko@tsurumi-u.ac.jp (R.O.); hanada-n@tsurumi-u.ac.jp (N.H.); 3 Chubu Kyodo Hospital, 3-20-1 Misato, Okinawa 904-0011, Japan; cyukyousika@yahoo.co.jp; 4 Department of Oral Microbiology, School of Dental Medicine, Tsurumi University, Yokohama 2308501, Japan; kakuta-erika@tsurumi-u.ac.jp; 5 Department of Operative Dentistry, School of Dental Medicine, Tsurumi University, Yokohama 2308501, Japan; okada-a@tsurumi-u.ac.jp; 6 Division of General Dentistry, Kyushu University Hospital, Kyushu University, Fukuoka 8128582, Japan; iyuko@dent.kyushu-u.ac.jp; 7 The LION Foundation for Dental Health, Tokyo 1308644, Japan; nori@pm-ms.tepm.jp

**Keywords:** mucosal brushing, oral care, C-reactive protein, body temperature, mixed effect model

## Abstract

This study was based in a hospital setting. Patients with acute symptoms face a life-threatening crisis and often have systemic complications during the convalescence stage. During the acute stage, oral function does not work and oral hygiene status deteriorates. A gauze or sponge brush is generally used to wipe the oral cavity; however, this process does not clean the oral cavity enough. Effective oral care requires better methods. Patients participating in this study were all hospitalized by ambulance and with acute symptoms. During the convalescence stage, patients were assigned application of mucosal brushing or wiping by gauze or sponge brush by order of hospitalization. The effects were evaluated by the number of bacteria on the tongue surface, serum C-reactive protein (CRP) and body temperature. Changes in bacterial count, body temperature, and CRP were effectively reduced in the mucosal brushing group compared to the wiping by gauze or sponge brush group. Based on mixed effect modeling, the coefficient of mucosal brushing for CRP was −2.296 and for body temperature was −0.067 and statistically significant. This simple method can effectively prevent systemic complication of inpatients with deteriorated oral conditions. This method may also be effective for the elderly in nursing homes or perioperative oral-care management.

## 1. Introduction

Oral-care management plays an important role in the prevention of systemic complications arising in hospitalized patients, especially pneumonia [1,2,3]. Patients with acute symptoms face a life-threatening crisis. At this stage, oral function does not work and oral hygiene status deteriorates. Even dental hygienists or nurses cannot apply oral care at this stage. Therefore, oral-care management during the convalescence stage is important for the prevention of systemic complications including pneumonia or pyrexia.

Dysphagia and oral health are the major complications leading to pneumonia [4,5,6,7], especially for the patients with neurological disorders pertaining dysphagia issue. Oral health and stroke were related [8,9]. Oral health were poor in patients with acquired brain injury and oral hygiene interventions improve the oral health status [10].

Previous reports have shown that some types of oral microbiome profiles on the tongue surface are at risk of post-operative pneumonia [11,12,13,14]. Cleaning of the oral cavity is indispensable for the prevention of systemic complications for hospitalized patients. Therefore, oral-care management has been introduced into the Japanese national insurance system. Nurses or, in some cases, dental hygienists clean the oral cavity of hospitalized patients using a gauze or sponge brush. However, wiping out of oral mucosa by gauze or sponge brush may not be enough. They cannot clean the lingual papillae of the tongue completely because of its complex form. By using a conventional toothbrush, the reduction of the bacterial load on the tongue surface was found to be negligible [15,16].

It has been suggested that oral care has another important role in the stimulation of oral mucosa. Brushing the teeth and mucosa lead to the stimulation of the salivary gland and promote the flow of saliva [17]. For this purpose, a robust and large brushing head is desirable for mucosal cleaning. Mucosal brushes have a large and robust head which is useful for stimulation of overall oral mucosa and effective for cleaning the tongue surface. Using a mucosal brush as part of oral-care management may be effective for the prevention of systemic complications and the recovery of oral functions.

The aim of this study was to evaluate the effect of mucosal brushing on oral bacterial levels, incidence of fever and serum C-reactive protein (CRP) that reflects a systemic inflammation status. In this study, we evaluated the effect of mucosal brushing for patients hospitalized in acute care in the convalescence stage.

## 2. Methods

### 2.1. Subjects and Setting

Patients who were hospitalized in a medical ward of internal medicine with acute symptoms between August 2019 and December 2019 were investigated. In total, 18 subjects agreed to participate in this study. One subject who was assigned to the gauze or sponge brush group missed measurement of CRP at baseline. This subject was excluded from analysis. The internal medicine wards mainly admit older patients, and many of them have dementia. For these patients, centralized management is necessary to manage not only the disease but their behavior. Many of them cannot brush their teeth by themselves, and need to be separated from other patients. Dental hygienists are stationed in these wards. The study population consisted of 2 men and 15 women; their mean age was 89.61 +/− 6.72 (74–97).

### 2.2. Study Design

Oral-care intervention, including oral examination, was started after acute symptoms such as pyrexia or vomiting had disappeared. A decision was made by a medical doctor.

Data concerning systemic conditions were collected from routine medical examination and medical records: Body temperature, serum CRP, original diseases for hospitalization, feeding conditions, and medications.

### 2.3. Oral Examination

Oral examination was carried out by one dentist. Number of remaining teeth and denture use were recorded. Additionally, the number of total bacteria on tongue surface and wettability of saliva were measured by a dental hygienist under the instruction of the dentist.

Oral bacterial were counted by using a Bacterial Counter (PHC Corporation, Tokyo, Japan) according to the manufacturer’s instructions [18]. This device counts total bacteria and does not distinguish between aerobe and anaerobe. Samples were obtained by swabbing the dorsum of the tongue three times by cotton swab. The cotton swab was immersed in the specialized liquid for this device. The impedance of the liquid was measured and the data were automatically transformed to cfu/mL in this device.

Patients’ level of dysphagia was evaluated by the Dysphagia Severity Scale rating. Slight injury: Oral problems, Minimum problems; Slander: Water aspiration, Occasional aspiration; Severe: Food aspiration, Saliva aspiration [19].

Wettability of saliva was evaluated by scaled tear production measuring strips (Schirmer: AYUMI Pharmaceutical Corporation, Tokyo, Japan) [20]. Strips were put on the tongue for 10 s and the soaked length was measured. These measurements were carried out at 8:00 a.m. before breakfast by one dental hygienist under the instruction of the dentist. Patients had their meal by tube feeding or oral ingestion. All the subjects who participated in this study needed help with meals by oral ingestion. None could eat their meal independently.

### 2.4. Oral Hygiene Procedures Implemented by Nurse

For the control groups, teeth were brushed with the patients’ own conventional toothbrush. Oral mucosa and teeth were wiped by an absorbent gauze or sponge brush (Oral care swab, Halyard Health, Inc., Tokyo, Japan). For mucosal brushing groups, a mucosal brush (ERAC 541 S: Lion COLTD, Tokyo, Japan) was used for brushing the teeth and oral mucosa. This mucosal brush was specially design for nursing care. The bristles were soft and the number of bristles was 48. Remnants on the brush were washed by water and any residual water in the brush was removed by gauze. These procedures were carried out by a nurse within 1 min once a day. For denture users, the denture was brushed under flowing water once a day. The denture was removed during sleep. These procedures were carried out from 10:00 a.m. Assignment of mucosal brushing or wiping by gauze or sponge brush were decided by the order of hospitalization. Sequential photos and a video of the mucosal brushing are presented in Supplementary Materials.

### 2.5. Statistical Analysis

To compare the baseline characteristics between the mucosal brushing group and the control group, Mann–Whitney U tests were applied. For the dichotomous variable, Fisher’s exact tests were used. To predict the changes in serum levels of CRP, mixed effect modeling was applied [21,22,23]. The model was specified by the following formula.

(1) $$\begin{array}{ll} -L1:\mathrm{CRP}=\pi_{0jk} & +\pi_{1ij}{\left(Age\right)}_{ij}+\pi_{2ij}{\left(Sex\right)}_{ij}+\pi_{3ij}{\left(Intervention\right)}_{ij}\\ & +\pi_{4ij}{\left(Number\;of\;remaining\;teeth\right)}_{ij}\\ & +\pi_{5j}{\left(Tube\;feeding/Oral\;ingestion\right)}_{ij}+\pi_{6,7j}{\left(medication\right)}_{ij}\\ & +\varepsilon_{ij} \end{array}$$

(2) $$\begin{array}{ll} -L2:\pi_{0jk}=\beta_{00k}^{\left(m\right)} & +\overset{17}{\underset{m=1}{\sum }}\beta_{001}^{\left(m\right)}{\left(days\;after\;hospitalization\right)}_{j}\\ & +\overset{17}{\underset{m=1}{\sum }}\beta_{001}^{\left(m\right)}{\left(days\;after\;oral\;care\;start\right)}_{j}r_{0j} \end{array}$$

(3) $$e_{ij}\sim N\left(0,\delta_{e}^{2}\right),r_{oj}\sim N\left(0,\delta_{r}^{2}\right)$$

Fixed effect: age, sex, days, intervention, number of remaining teeth, tube feeding/oral ingestion, days after hospitalization, medication
Random effect: days after hospitalization
Covariance Type: AR1
Link functions: normal

Analysis was performed using SPSS Statistics version 24.0 (IBM, Tokyo, Japan).

### 2.6. Ethics

Informed written consents were obtained from all of the subjects participating in this study after an explanation of the aim of this study by the dentist. The Ethics Committee of Okinawa Kyodo Hospital approved this study (approval number: 2019-003, approved date: 20 May 2019), in accordance with the declaration of Helsinki.

## 3. Results

### 3.1. Characteristics of the Subjects Participating in this Study

After improvement of the acute symptoms, oral examinations and oral care were applied. Mean duration after initial hospitalization was 6.35 +/− 6.32 days. The baseline characteristics of the patients are shown in Table 1. No statistically significant differences were observed between the two groups. Original diseases causing hospitalization and requiring medication are shown in Table 2 and Table S1.

### 3.2. Changes of Bacteria on Tongue Surface

**Figure 1:**
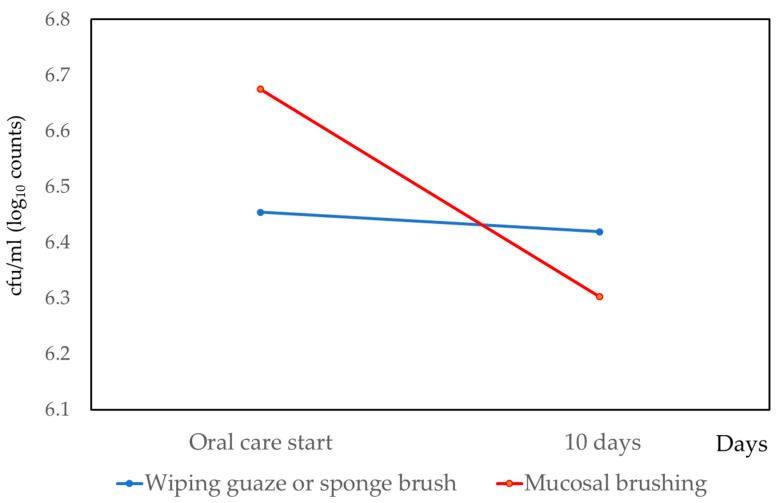
Changes of oral bacterial levels by conventional oral care and mucosal brushing.

Bacteria on the tongue surface were measured on the day that oral-care management started, and after 10 days. The results are shown in Figure 1. Bacteria on the tongue surface were reduced in both the gauze or sponge brush group and the mucosal brushing group. The differences between the two groups were statistically significant (*p* < 0.001).

For the mucosal brushing group, the number of bacteria on the tongue surface were effectively reduced. The difference was statistically significant (*p* < 0.001).

### 3.3. Changes of CRP and Body Temperature

**Figure 2:**
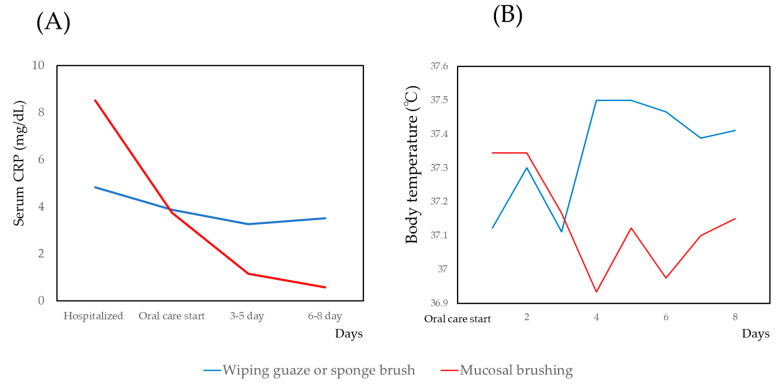
Changes in C-reactive protein (CRP) and body temperature with conventional oral care and mucosal brushing. (**A**)Serum CRP, (**B**) Body temperature

The changes in the serum CRP levels and body temperature are shown in Figure 2. For the serum CRP and body temperature, the mixed effect model was applied. The results are shown in Table 3. The coefficient of intervention (mucosal brushing/conventional oral care) was statistically significant for both the serum CRP and body temperature. Other factors—age, number of remaining teeth, tube feeding/oral ingestion, days after hospitalization—were also statistically significant for CRP. However, the coefficient of intervention was largest.

For the mucosal brushing group, the number of bacteria on the tongue surface were effectively reduced.

## 4. Discussion

In this study, we confirmed the effect of mucosal brushing for the systemic inflammation evaluated by CRP and body temperature. There may be two pathways for the effect of mucosal brushing.

First, there are many lingual papillae on the dorsum of the tongue which broaden its surface area. On this surface, there exist a vast number of bacteria, including anaerobic bacteria [24]. There exist etiological bacteria for pneumonia. Oral health care is necessary for the treatment and prevention of repeated episodes of pneumonia in elderly patients [25]. Previous reports have shown that bacterial flora in the dorsum of the tongue affect the incidence of pneumonia in older people [26,27,28,29,30]. Oral bacteria count is significantly associated with the onset of pneumonia [31].

Oral bacteria are significantly reduced by tongue scrapping [32]. The tongue surface can be wiped by gauze or sponge brush; however, it cannot be scrubbed by a gauze or sponge brush. Using a mucosal brush can scrub the lingual papilla and remove the accumulated bolus of bacteria. The difference in cleaning efficiency using this method may be attributed to the reduction of pathogenic bacteria in the oral cavity. A previous study has shown that preoperative oral care can decrease inflammation during the early postoperative stage, and the CRP level in the early postoperative period was lower in the oral care group than in the non-oral care group [33]. Oral bacteria may accumulate not only on the tongue surface but also on other oral mucosa. Oral mucosa other than on the tongue were cleaned with a mucosal brush for patients in the mucosal brushing group. The difference in efficacy of reducing bacteria may be derived from this effect.

Subjects investigated in this study were patients who were institutionalized by emergency transport. For these subjects, implementation of oral care intervention was impossible during the acute stage. The number of bacteria on the tongue surface is known to increase after 2 or 3 days following surgical operation [33,34,35]. The oral condition of patients at the acute stage may be similar to that of patients immediately after surgical operation. CRP values were decreased after the start of oral care in both the mucosal brushing group and the gauze or sponge brush group. Efficiently reducing oral bacteria by mucosal brushing may lead to reduced inflammation and an improvement in CRP levels. Oral candidiasis is a risk of pneumonia and fevers [22], it may delay rehabilitation of dysphagia [36]. It is necessary for the evaluation of mucosal brushing for the deduction of oral fungi. It is one of the limitation of this study.

Secondly, a large and robust head on the mucosal brush is advantageous for the training of labial and tongue pressure. Mucosal brushing may lead to rehabilitation training of the oral function. In this study, training for swallowing function was not implemented. Previous reports have shown that the reinforcement of tongue pressure may lead to an improvement of the swallowing function, and prevention of pneumonia and chest infections [37,38]. Improvement of the swallowing function may prevent aspiration of oral bacteria, including pathogenic bacteria.

Reduction in oral bacterial numbers was ephemeral when using mechanical tongue cleaning [31]. Wiping by sponge or gauze can loosely approach the mucosal surface. Therefore, a reduction of oral bacterial may be limited. In contrast, mucosal brushing can fully approach the oral mucosa and effectively clean the surface of the tongue. The bacterial reduction may ultimately lead to an improvement in CRP levels.

There are several trials to evaluate oral health intervention method. Outcome variables of these studies were plaque and gingival bleeding control [39] and reduction of oral opportunistic pathogens [40]. It is difficult to compare the effect of mucosal brushing with these studies. However, powered tooth brushing, mouth rinsing with chlorhexidine applied these studies may be also effective.

The main limitation of this study was the small sample size. However, despite the small sample size, the effect of mucosal brushing was confirmed. Oral disease indexes such as DMF and periodontal indexes were not measured. For the patients analyzed in this study, these oral examinations were almost impossible. To evaluate these indexes, the study population needs to include more slightly injured patients. Effects of saliva flow [41,42] or other parameters can be evaluated.

In this study, we examined the effect of mucosal brushing for the improvement of body temperature and CRP levels. Further study is needed to elucidate the precise mechanism of the effect of mucosal brushing. However, this simple method can effectively prevent systemic complication of inpatients with acute symptoms.

## Figures and Tables

**Table 1:** Baseline characteristics of the subjects participated in this study.

	Gauze or Sponge Brush (*n* = 8)	Mucosal Brushing (*n* = 9)	Total (*n* = 17)	*p*-Value
Age	87.22 +/− 8.41	92.00 +/− 3.54	89.61 +/− 6.72	0.249
Men/Women	1/7	1/8	2/16	0.765
Tube feeding/oral ingestion	2/6	5/4	7/10	0.201
Denture use	5	5	10	0.722
Number of remaining teeth	2.22 +/− 3.35	0.79 +/− 1.39	1.50 +/− 2.60	0.451
Serum levels of CRP (mg/dL) (day of hospitalization)	3.12 +/− 4.29	4.89 +/− 5.41	3.95 +/− 4.78	0.700
Body temperature (°C)				
Day of hospitalization	34.04 +/− 10.3	37.23 +/− 0.45	35.54 +/− 7.47	0.961
Day of oral care start	37.12 +/− 0.25	37.24 +/− 0.44	37.18 +/− 0.36	0.504
Oral bacteria (log_10_ cfu)	4.26 +/− 2.7	5.81 +/− 2.46	5.04 +/− 2.63	0.233
Dysphagia Slight injury/Slander/Severe	1/6/1	1/4/4	2/10/5	0.620
Wettability of saliva	0.89 +/− 1.62	1.11 +/− 1.83	1.00 +/− 1.68	0.920

Patients’ level of dysphagia was evaluated by the Dysphagia Severity Scale rating. Slight injury: Oral problems, minimum Problems; Slander: Water aspiration, occasional aspiration; Severe: Food aspiration, saliva aspiration. *p*-values were calculated by Mann–Whitney U tests or Fisher’s exact tests.

**Table 2:** Original diseases causing hospital admission.

	Gauze or Sponge Brush (*n* = 8)	Mucosal Brushing (*n* = 9)
Aspiration pneumonia	4	4
Acute pyelonephritis	1	2
Urinary tract infection (UTI)	1	1
Cholangitis	1	0
Epilepsy, convulsive seizure	1	0
Aphagia	0	1
Hypernatremia, dehydration	0	1

**Table 3:** Mixed effect model analysis for the serum CRP and body temperature.

	CRP		Body Temperature	
	Coefficient	*p*-Value	Coefficient	*p*-Value
Intercept	−32.746 (−37.765–−27.727)	<0.001	36.900 (35.966–37.834)	<0.001
Age	0.357 (0.300–0.414)	<0.001	0.004 (−0.007–0.015)	0.455
Sex (Man/Woman)	2.911 (−0.0560–5.879)	0.054	−0.478 (−0.739–−0.216)	<0.001
Intervention (Mucosal brushing/Wiping gauze or sponge brush)	−2.296 (−4.486–−0.107)	0.040	−0.067 (−0.068–−0.065)	<0.001
Number of remaining teeth	0.887 (0.270–1.504)	0.006	0.006 (−0.030–0.041)	0.751
Tube feeding/Oral ingestion	−0.213 (−2.276–1.849)	0.835	−0.024 (−0.200–0.152)	0.787
Days after hospitalization	0.095 (0.095–0.095)	<0.001	0.001 (−0.015–0.017)	0.874
Medication (Antipyretic analgesic)	−1.901 (−4.206–0.404)	0.103	0.255 (−0.450–−0.060)	0.011
Medication (Antibiotic)	4.101 (3.873–4.329)	<0.001	0.120 (−0.143–0.382)	0.370
BIC	320.096		594.087	
AICC	363.105		423.837	

For the changes in serum CRP, intervention (mucosal brushing/wiping gauze or sponge brush) was statistically significant. Tube feeding indicates a nasogastric tube. A percutaneous endoscopic gastrostomy tube was not inserted into any of the patients. BIC: Bayesian information criterion. AICC: Akaike’s information criterion correction.

## Supplementary Materials

The following are available online at https://www.mdpi.com/1010-660X/56/10/549/s1, Table S1. List of medications of the subjects who participated in this study. Appendix 1. Sequential photos of the procedures of mucosal brushing. Appendix 2 and 3. Video of procedure of mucosal brushing.
