# Supplementary material for: Effect of Mucosal Brushing on the Serum Levels of C-Reactive Protein for Patients Hospitalized with Acute Symptoms
Source: Medicina (Kaunas). 2020 Oct 19;56(10):549. doi: 10.3390/medicina56100549 (PMC7603140; doi:10.3390/medicina56100549)
Supplement: Supplementary file 1 [file medicina-56-00549-s001.zip › medicina-953252-SI/Supplimetal Ver3/Appendix1.pptx]

## Slide 1
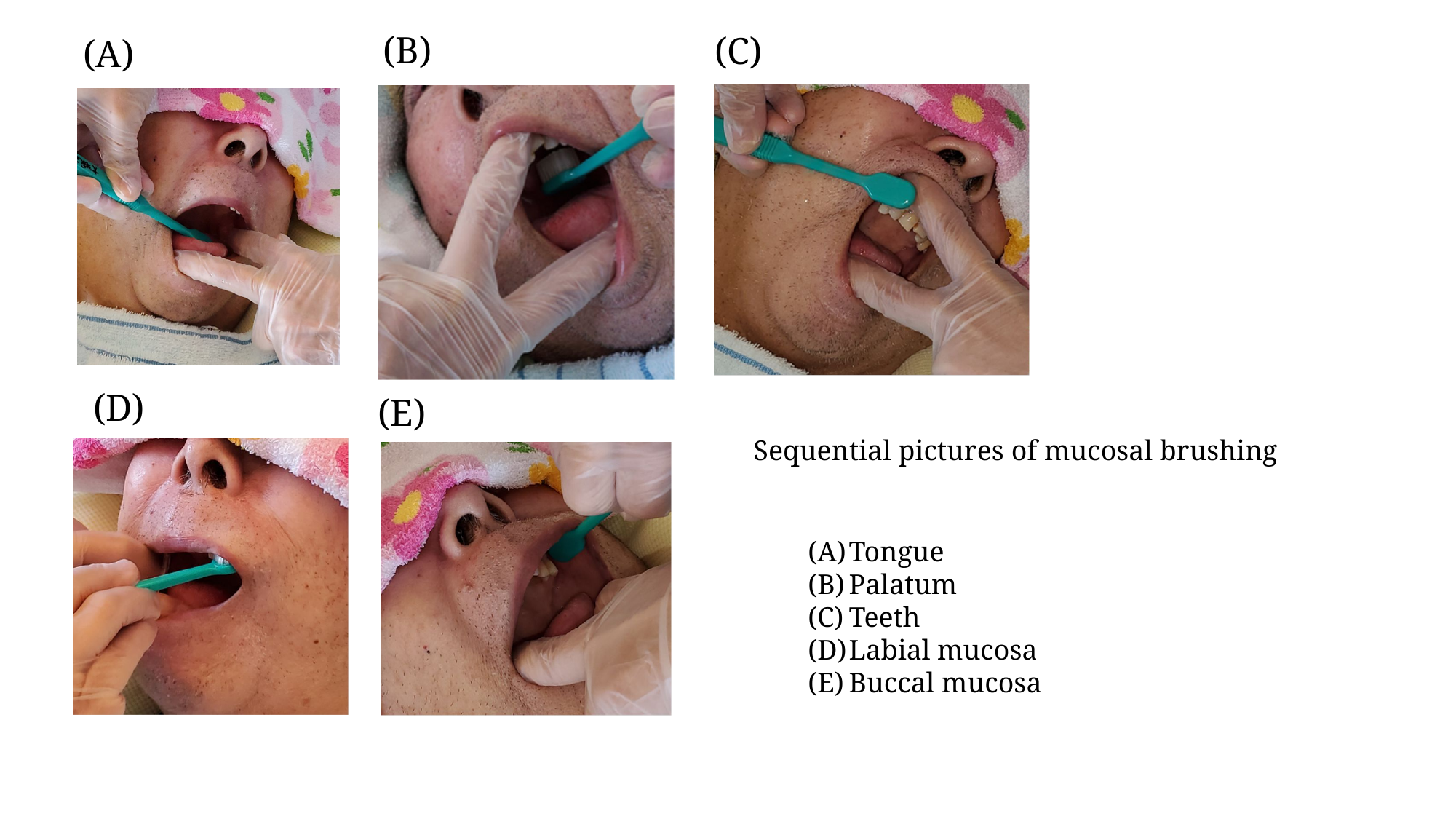

(B)
(C)
(A)
(D)
(E)
Sequential pictures of mucosal brushing
Tongue
Palatum
Teeth
Labial mucosa
Buccal mucosa
