# Supplementary material for: Effect of Mucosal Brushing on the Serum Levels of C-Reactive Protein for Patients Hospitalized with Acute Symptoms
Source: Medicina (Kaunas). 2020 Oct 19;56(10):549. doi: 10.3390/medicina56100549 (PMC7603140; doi:10.3390/medicina56100549)
Supplement: Supplementary file 1 [file medicina-56-00549-s001.zip › medicina-953252-SI/Supplimetal Ver3/medicina-953252-SI.pdf]

medicina

MDPI

Supplemental Materials

Table S1. List of medications of the subjects who participated in this study.

	Mucosal brushing	Conventional oral care	Total
Antibiotic	7	6	13
Vasodilator	3	7	10
Antiulcer agent	4	5	9
Cathartic	5	4	9
Vitamins	3	6	9
Antipyretic analgesic	2	4	6
Antipsychotic	3	3	6
Analeptic	3	2	5
Antihypertensive	4	1	5
Diuretic	1	3	4
Expectorant	1	2	3
Anticoagulant	1	2	3
Hormone preparation	2	1	3
Hemostatic	2	1	3
Spasmolytic	1	1	2
Antiparkinsonian	2	0	2
Central nervous system depressant	1	1	2
Antifolate	1	0	1
Antineoplastic	1	0	1
Lipid-lowering drug (Dehydration)	0	1	1
Hypnotic	0	1	1
Anxiolytic	0	1	1
Chemotherapeutic	0	1	1
Autonomic agen	1	0	1
Antiepileptiv	0	1	1
Purified glucose	3	3	6
Sodium chloride	6	6	12

Herbal medicine	2	3	5
Agents affecting digestive organs	1	0	1
